# A multiplexed targeted method for profiling of serum gangliosides and glycosphingolipids: application to GM2-gangliosidosis

**DOI:** 10.1007/s00216-024-05487-3

**Published:** 2024-08-27

**Authors:** Jinyong Kim, Seul Kee Byeon, Devin Oglesbee, Matthew J. Schultz, Dietrich Matern, Akhilesh Pandey

**Affiliations:** 1 Department of Laboratory Medicine and Pathology, Mayo Clinic, Rochester, MN USA; 2 Center for Individualized Medicine, Mayo Clinic, Rochester, MN USA; 3 Manipal Academy of Higher Education, Manipal, Karnataka India

**Keywords:** Lipidomics, Gangliosides, Glycosphingolipids, GM2-gangliosidosis, Multiplexed, LC–MS/MS

## Abstract

The analysis of gangliosides and glycosphingolipids is crucial for understanding cellular membrane structure and function as well as to accurately diagnose certain inborn errors of metabolism. GM2-gangliosidosis represents a rare and fatal group of lysosomal storage disorders characterized by accumulation of GM2 gangliosides in various tissues and organs. These disorders arise due to deficiency or functional impairment of the β-hexosaminidase A or B enzymes, which are responsible for degradation of GM2 ganglioside. Deficient enzyme activity primarily leads to the accumulation of GM2 gangliosides within the lysosomes of cells. Accurate and rapid diagnostic methods that detect increased levels of GM2 gangliosides in patients with GM2-gangliosidosis can play a significant role in early diagnosis and appropriate treatment of this condition. To address this need, we developed a multiplexed liquid chromatography-tandem mass spectrometry method targeting 84 species of gangliosides and other glycosphingolipids involved in ganglioside metabolism. Reproducibility, linearity, extraction efficiency, and sample stability were evaluated and proof-of-concept data obtained from analysis of serum samples from confirmed cases of GM2-gangliosidosis. This method has the potential to simultaneously monitor the biosynthesis of gangliosides and the lysosomal catabolic pathway serving as a valuable tool for screening and diagnosing an important group of lysosomal storage disorders.

**Supplementary Information:**

The online version contains supplementary material available at 10.1007/s00216-024-05487-3.

## Introduction

Gangliosides are a type of glycosphingolipids that are sialylated and consist of a hydrophobic ceramide and an oligosaccharide chain. The diverse sugar composition, number of sialic acids, and variation in the length of carbon chains of ceramide result in over 200 distinct molecular species of gangliosides [1]. These molecules form functional microdomains, known as lipid rafts, on cell membranes by associating laterally with sphingomyelin, cholesterol, and proteins owing to the hydrophobic nature of their tails and the polar glycans [2]. Gangliosides are predominantly found in human brain tissue, where they play vital roles in myelin maintenance, cell signaling, and cell–cell interactions [3–5]. The synthesis of gangliosides occurs in the endoplasmic reticulum and Golgi apparatus while their degradation takes place in the lysosome. The degradation of glycosphingolipids involves β-hexosaminidases A and B, which consist of either α and β subunits or β and β subunits, respectively. Specifically, β-hexosaminidase A, aided by a protein called GM2 Activator Protein (GM2AP), catalyzes the conversion of GM2 ganglioside to GM3 ganglioside [6].

GM2-gangliosidosis is a rare and devastating group of inherited lysosomal storage disorders characterized by the accumulation of GM2 ganglioside in various tissues and organs. These disorders result from a deficiency or dysfunction of β-hexosaminidase A or B enzymes, which are responsible for the degradation of GM2 ganglioside. GM2-gangliosidosis encompasses three distinct phenotypes: Tay-Sachs disease, Sandhoff disease, and the AB variant [7, 8]. Each phenotype is associated with specific genotype affecting the genes encoding the α and β subunits of the β-hexosaminidase enzyme complex. The deficient enzyme activity leads to the accumulation of GM2 ganglioside primarily within the lysosomes of cells. GM2-gangliosidosis predominantly affects the central nervous system resulting in progressive neurodegeneration. Infants affected by the disease appear normal at birth but gradually develop developmental regression, motor impairment, and intellectual disability. Progression is characterized by the deterioration of cognitive function, loss of motor skills, seizures, and eventual paralysis. Other symptoms may include visual and auditory impairment, respiratory difficulty, and organ dysfunction [9–11].

The current diagnosis of GM2-gangliosidosis is typically performed through clinical evaluation and biochemical testing. GM2-gangliosidosis commonly arises from deficiency in β-hexosaminidase A or B enzymes. Thus, the diagnosis is often based on measuring β-hexosaminidase enzyme activity in blood, bone marrow, or other relevant samples [10–12]. Generally, patients with GM2-gangliosidosis exhibit significantly reduced or absent enzyme activity. However, while β-hexosaminidase enzyme activity measurement aids in a rapid and relatively cost-effective diagnosis of GM2-gangliosidosis, certain genotypes may result in enzyme activity being within the normal range leading to false-negative results. Genetic testing performed to diagnose GM2-gangliosidosis by identifying pathogenic variants in the *HEXA* or *HEXB* is the most definitive and accurate diagnostic method but can be costly and time-consuming. However, even with genetic testing, the possibility of novel pathogenic variants cannot be ruled out [13].

Quantitative analysis of GM2 ganglioside and related molecules in blood, urine, cerebrospinal fluid, and other relevant samples can provide a sensitive and precise approach for the diagnosis of GM2-gangliosidosis [11, 12, 14–20]. By measuring the levels of GM2 and other gangliosides including GM3 gangliosides or other glycosphingolipids, clinicians can assess the accumulation of these analytes that are indicative of GM2-gangliosidosis. Various analytical techniques, including enzyme-linked immunosorbent assays (ELISA) [21, 22], ion mobility mass spectrometry [23], and liquid chromatography tandem mass spectrometry (LC–MS/MS) [14, 16–19], have been utilized for this purpose. Highly sensitive LC–MS/MS technology has recently emerged as an effective tool for characterizing the alteration of gangliosides in GM2-gangliosidosis although a full characterization of structural complexity of gangliosides remains to be addressed.

In this study, we developed a multiplexed LC–MS/MS method targeting a total of 84 species of gangliosides and glycosphingolipids from serum samples. This method targets 10 classes of gangliosides (GM1, GM2, GM3, GD1, GD2, GD3, GT1, GT2, GT3, and GQ1) and 4 classes of glycosphingolipids (GlcCer, LacCer, Gb3, and GA2) by alternating between positive and negative ion mode detection in a single mass spectrometry run. This method allows for the monitoring of the entire pathway of ganglioside metabolism. Using this approach, we monitored alteration of individual target species in serum samples from patients with GM2-gangliosidosis including Tay-Sachs and Sandhoff disease. This multiplexed LC–MS/MS method holds promise for the diagnosis and screening of various metabolic disorders involving gangliosides and other glycosphingolipids.

## Materials and methods

### Materials and reagents

The following standards were purchased: GM3 (d18:1/18:0), GD3 (d18:1/18:0), GD1a (d18:1/18:0), GT1b (d18:1/18:0), glucosyl ceramide (d18:1/16:0), lactosyl ceramide (d18:1/18:0 and d18:1/24:0), and Gb3 (d18:1/18:0) from Avanti Polar Lipids (Alabaster, AL, USA); GM2 (d18:1/18:0) and GM1 (d18:1/18:0) gangliosides from Cayman Chemical (Ann Arbor, MI, USA); GD2 (d18:1/18:0) ganglioside from ChemCruz (Dallas, TX, USA). Glycosphingolipids (Avanti Polar Lipids Inc., Alabaster, AL, USA) with odd-numbered or deuterated acyl chain were used as internal standards and were added to the serum lipid extracts for multiple reaction monitoring (MRM)–based quantitation: GM3 (d18:1/18:0-d_5_), GM1 (d18:1/17:0), glucosyl ceramide (d18:1/16:0-d_3_), and Gb3 (d18:1/18:0-d_3_). High-performance liquid chromatography (HPLC)–grade solvents (water, acetonitrile, methanol, isopropanol, and chloroform) were purchased from Fisher Scientific (Waltham, MA, USA).

### Human serum samples

As a proof-of-concept, samples from confirmed cases of patients with GM2-gangliosidosis were analyzed. Serum samples from two patients with Tay-Sachs disease, a patient with Sandhoff disease, and 36 unaffected controls were obtained after written consent. Age and sex of controls and patients are described in Supplementary Table 1. This study was approved by the institutional review board (IRB protocol 21–012890) of Mayo Clinic in accordance with the Declaration of Helsinki.

### Sample preparation

For lipid extraction [24], 20 µl of serum was mixed with deuterated standard mixture as an internal standard (IS; GM3-d_3_ d18:1/18:0, GM1 d18:1/17:0, GlcCer-d_3_ d18:1/16:0, and Gb3-d_3_ d18:1/18:0), followed by addition of 200 µl of chloroform:methanol (1:2, v/v). The samples were vortexed briefly and incubated in a bath sonicator for 30 min. After centrifugation, supernatant was collected and dried under pure nitrogen. The dried lipid extract was reconstituted in 20 µl of methanol and mixed before transferring to autosampler vials. A standard mixture was prepared by mixing 5 pmol/µl of the following standards: GM3 d18:1/18:0, GM2 d18:1/18:0, GM1 d18:1/18:0, GD3 d18:1/18:0, GD2 d18:1/18:0, GD1a d18:1/18:0, GT1b d18:1/18:0, GlcCer d18:1/16:0, LacCer d18:1/18:0, LacCer d18:1/24:0, and Gb3 d18:1/18:0. This standard mixture was diluted serially to generate a calibration curve by spiking into serum substitute matrix.

### LC–MS/MS

The targeted LC–MS/MS analysis was carried out on a QTRAP 6500 + mass spectrometer (Sciex, Framingham, MA) connected to an LC 400 system with microflow module (5 to 50 µl/min). Separation of gangliosides was performed according to their number of sialic acid and carbon chain length in target lipids using phenyl-hexyl column (Waters BEH phenyl; 1.0 × 50 mm, 1.7 µm) with a flow rate of 20 µl/min for 15 min using a binary gradient of mobile phase A (water:acetonitrile (9:1, v/v) with 5 mM ammonium hydroxide and 1 mM ammonium formate) and mobile phase B (isopropanol:methanol:acetonitrile (7:1.5:1.5, v/v/v) with 5 mM ammonium hydroxide and 1 mM ammonium formate). Five microliters of samples was injected into the analytical column with 10% of mobile phase B for 1 min. After sample loading, mobile phase B was increased to 70% over 1 min, 100% over 4 min, and maintained at 100% for another 5 min. Thereafter, the mobile phase B was decreased to 10% and the analytical column was reconditioned for 4 min. MRM analysis was performed in a positive/negative ion switching mode. MS parameters in the multiplexed MRM method are listed in Supplemental Table 2. In all, 84 molecular ions monitored for gangliosides (GM3, GM2, GM1, GD3, GD2, GD1, GT3, GT2, GT1, and GQ1) and glycosphingolipids (GlcCer, LacCer, Gb3, and GA2) in a single run using scheduled MRM acquisition with 1-min retention time window (Supplemental Table 2). All targeted lipids were quantified by calculating the peak areas of extracted ion chromatograms, followed by normalization with reference to peak area of internal standards.

### Method validation

The standard mixture spiked into serum substitute was used for obtaining calibration curve as well as limit of detection and limit of quantification. The linearity of the method was evaluated using calibrators across different concentration ranges. The linearity equation and coefficient of determination (*r*^2^) were determined based on a calibration curve. The limit of detection was calculated based on the standard deviation of the response (Sy) of the calibration curve and the slope of the calibration curve (S) at levels approximating the LOD according to the formula: LOD = 3.3(Sy/S). Intra-assay reproducibility and inter-assay reproducibility were assessed by spiking low (low QC, 50 fmol/µl) and high concentration (high QC, 1 pmol/µl) standard mixture into 20 µl of serum substitute supplement. Intra-assay reproducibility was evaluated by running the low and high QC samples five times in a day. Inter-assay reproducibility was evaluated by running the low and high QC samples five times daily over 5 days. Extraction efficiency was evaluated by comparing the abundance levels of the high concentration standards (1 pmol/µl) described above that were added to the serum substitute prior to lipid extraction with those added after lipid extraction during the reconstitution step. By comparing the peak areas of extracted standards to equal amounts of standards that did not go through lipid extraction, extraction yield (%) for each standard was calculated as follows: (calculated amount of lipids spiked before extraction / calculated amount of lipids spiked after extraction) × 100%. The matrix effect was assessed by comparing the peak area of the low and high QC mixture spiked into a serum substitute to the peak area obtained from the neat standard mixtures without the matrix. Matrix factor was calculated as follows: (peak area in matrix / peak area in methanol) × 100%.

Sample stability was evaluated by aliquoting serum samples from five healthy donors and storing them at room temperature (18 to 25 °C; baseline, 1, 3, 7 days), refrigerated (4 °C; baseline, 1, 3, 7, 14 days), and frozen (− 20 °C; baseline, 1, 3, 7, 14 days). After each time point, the samples were frozen at − 80 °C. Subsequently, the samples were thawed and analyzed on the same day in the same set. Freeze–thaw stability was assessed over four freeze–thaw cycles. The stability of samples after lipid extraction was evaluated while stored in the autosampler at 4 °C over 48 h.

### Data analysis

All targeted glycosphingolipids were quantified using Skyline from precursor and quantifier ions [25]. The peak areas of the target lipids were normalized to the peak areas of internal standards. Owing to lack of commercially available deuterated standards for all target lipids, internal standards with the closest chemical structures were used for quantification. Significant differences in level of detected ganglioside and glycosphingolipid targets between GM2-gangliosidosis patients and control individuals were evaluated using two-tailed Student’s *t*-test. Grubb’s test was applied to remove any outliers from the dataset. The principal component analysis and heatmap were generated using MetaboAnalyst 5.0 (http://www.metaboanalyst.ca) [26] and box plots were visualized using BoxPlotR (http://boxplot.tyerslab.com).

## Results

### Development of a multiplexed method to detect gangliosides and glycosphingolipids

**Fig. 1:**
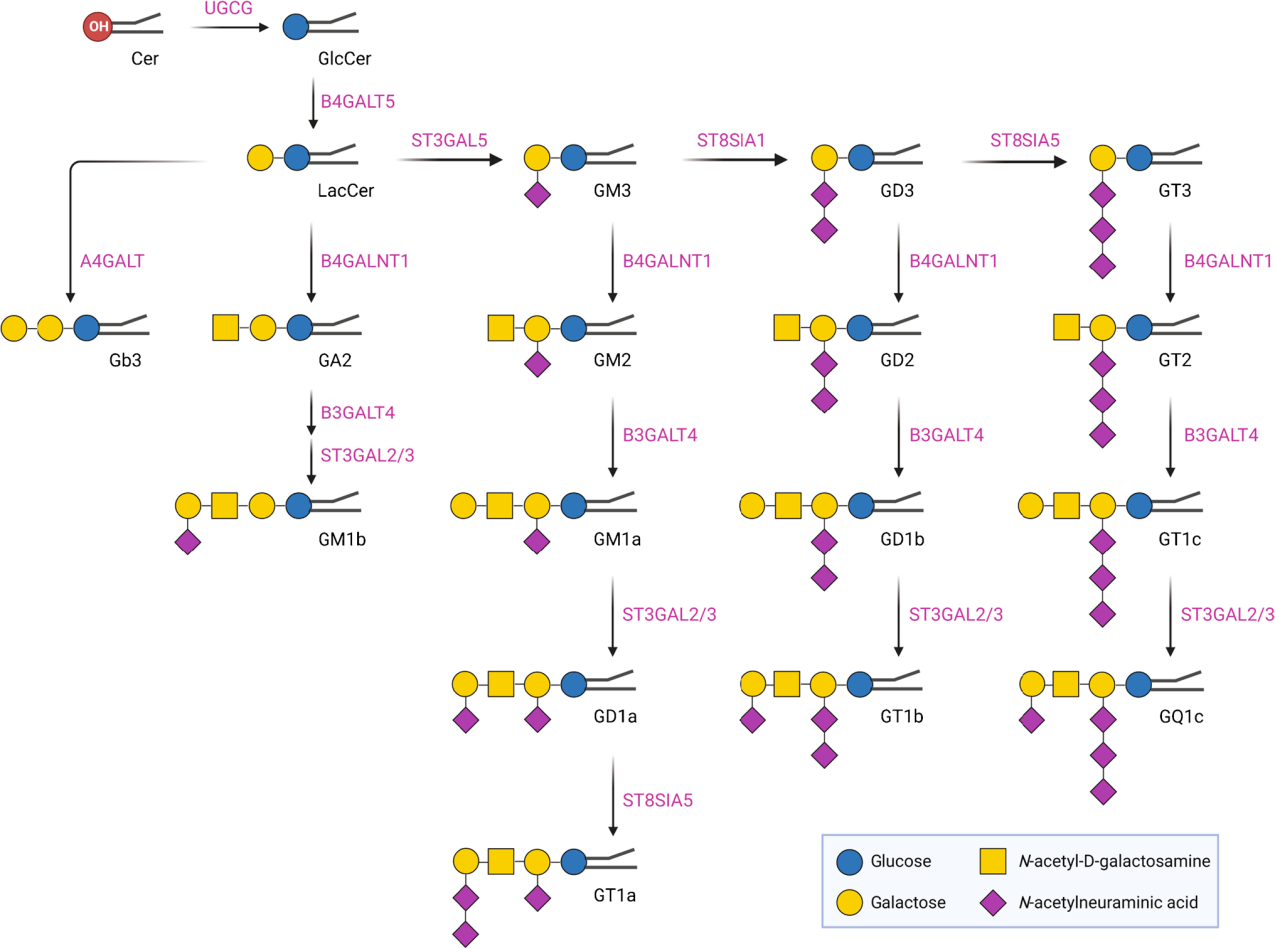
Biosynthesis pathway–associated various classes of gangliosides and glycosphingolipids targeted by the multiplexed LC–MS/MS method. Biosynthetic enzymes are labeled in pink. The abbreviations used for analytes and enzymes are as follows: Cer, ceramide; GlcCer, glucosyl ceramide; LacCer, lactosyl ceramide; Gb3, globo-triaosylceramide; GM1, monosialo-tetrahexosylganglioside; GM2, monosialo-trihexosylganglioside; GM3, monosialo-dihexosylganglioside; GD1, disialo-tetrahexosylganglioside; GD2, disialo-trihexosylganglioside; GD3, disialo-dihexosylganglioside; GT1, trisialo-tetrahexosylganglioside; GT2, trisialo-trihexosylganglioside; GT3, trisialo-dihexosylganglioside; GQ1, quadrisialo-tetrahexosylganglioside; GA2, asialo-trihexosylganglioside; UGCG, UDP-Glucose Ceramide Glucosyltransferase; B4GALT5, UDP-Gal:Beta-GlcNAc Beta-1,4-Galactosyltransferases 5; ST3GAL5, CMPNeuAc:Lactosylceramide Alpha-2,3-Sialyltransferase; ST8SIA1, ST8 Alpha-N-Acetylneuraminide Alpha-2,8-Sialyltransferase 1; ST8SIA5, ST8 Alpha-N-Acetyl-Neuraminide Alpha-2,8-Sialyltransferases 5; B4GALNT1, UDP-Gal:BetaGlcNAc Beta-1,4 N-Acetylgalactosaminyltransferase 1; B3GALT4, UDP-Gal:BetaGlcNAc Beta 1,3-Galactosyltransferase 4; ST3GAL2/3, CMP-N-Acetylneuraminate-Beta-Galactosamide-Alpha-2,3-Sialyltransferase 2; ST8SIA5, ST8 Alpha-N-Acetyl-Neuraminide Alpha-2,8-Sialyltransferase 5

**Fig. 2:**
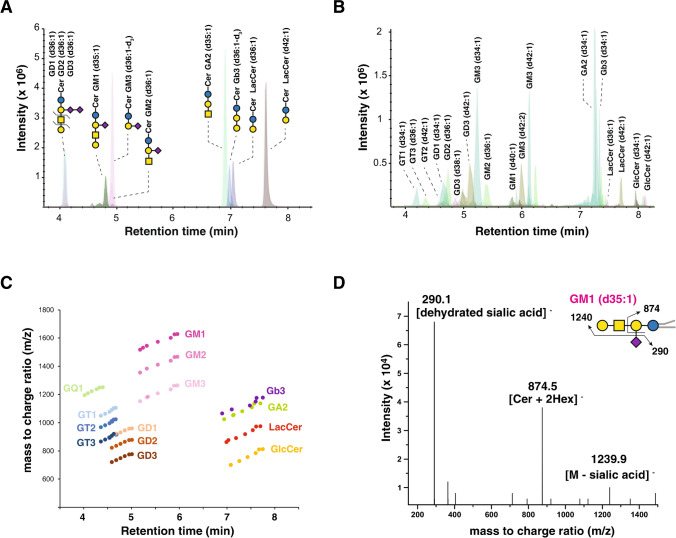
Detection of gangliosides and glycosphingolipids by a multiplexed LC–MS/MS method. **A** Separation of a mixture containing six ganglioside (GM1 d18:1/17:0, GM2 d18:1/18:0, GM3 d18:1/18:0, GD1 d18:1/18:0, GD2 d18:1/18:0, GD3 d18:1/18:0-d_3_) and four glycosphingolipid (LacCer d18:1/18:0, LacCer d18:1/24:0, Gb3 d18:1/18:0-d_3_, GA2 d18:1/17:0) standards using a phenyl-hexyl column. **B** Extracted ion chromatograms obtained from lipids extracted from normal serum sample targeting various species of gangliosides and glycosphingolipids. **C** Elution map displaying the retention times of the targeted species detected in normal serum samples. **D** MS/MS spectrum of GM1 ganglioside (d35:1)

Targeted analysis for serum gangliosides was performed in a 15-min LC–MS/MS method in MRM mode. Figure 1 shows the principal biosynthetic pathway of all targeted gangliosides and other glycosphingolipids in a multiplexed LC–MS/MS method. This multiplexed method applied to the standard mixture and human serum quantified a total of 84 species of GM3, GM2, GM1, GD3, GD2, GD1, GT3, GT2, GT1, and GQ1 gangliosides and other glycosphingolipids including GlcCer, LacCer, Gb3, and GA2 (Svennerholm nomenclature [27] was used). By using phenyl-hexyl HPLC column, the gangliosides were separated according to their number of sialic acids and, within a specific class of ganglioside, gangliosides possessing different ceramide carbon chain lengths were separated [28]. The performance of phenyl-hexyl column is demonstrated by the separation of mixture of six ganglioside and four glycosphingolipid standards (GM1, GM2, GM3, GD1, GD2, GD3, LacCer, Gb3, and GA2) as shown in Fig. 2A, which were obtained with 1 pmol of each standard. Figure 2B shows the extracted ion chromatograms obtained from lipid extracts of human serum sample by targeting all species of gangliosides and glycosphingolipids as depicted in Fig. 1. The elution map of the targeted species detected in normal serum samples is shown in Fig. 2C. Gangliosides belonging to classes with higher sialic acid content, GQ1, eluted first, while gangliosides with a single sialic acid, such as GM1, GM2, and GM3, eluted the latest among the gangliosides. Glycosphingolipids exhibited minor variations in retention times based on the number of glycans bound to ceramide. Within each class, differentiation based on fatty acyl chain length is possible using retention time. As the dehydrated sialic acid fragment (m/z 290.1) was observed as the most dominant product ion when gangliosides were fragmented in negative ion mode (Fig. 2D). Thus, the fragment ion at m/z 290 was chosen as the product and quantifier ion for all GM3, GM2, GM1, GD3, GD2, GD1, GT3, GT2, GT1, and GQ1 gangliosides detected in negative ion mode. Glycosphingolipids other than gangliosides were detected in the positive ion mode using fragment ions corresponding to dehydrated sphingosine backbone (m/z 264.3) as the product and quantifier ion.

The standard mixture was spiked into serum substitute matrix, which contains human serum albumin and globulins but not lipids [29], to generate a calibration curve as well as to determine the limit of detection (Table 1). To evaluate the efficiency of a single-phased extraction using chloroform:methanol (1:2, v/v) as the extraction solvent, the peak areas of analytical standards were compared before and after extraction when added to a serum substitute. The average extraction efficiency for all standards was above 90% with a median of 97.3% (Supplemental Table 3). The performance of calibration standards prepared at various concentrations was assessed by calculating the coefficients of determination (*r*^2^) from three replicate runs. All *r*^2^ values were above 0.99, with a median of 0.994 (Table 1). Intra-assay precision showed < 10% coefficient of variation for low and high concentration mixture. Inter-assay precision was performed by measurements a day across five days and was < 18% for both low and high QC mixture (Table 1). The serum matrix effect was evaluated using low and high QC samples, and the matrix factor for all standards was an average of 79.5% in the low QC mixture and 87.5% in the high QC mixture (Supplemental Table 4). The sample stability of targeted lipids was evaluated under three storage conditions. Samples were considered unstable if the relative recovery values changed by > 15% compared to the baseline (at day 0) results before storage. The stability of each lipid class in the samples was assessed by summing the quantification results of the targeted subspecies for each lipid class, using normalized peak areas with IS for comparison. Under room temperature conditions (18 to 25 °C), all lipid classes except GD1 and GT2 were stable for up to 3 days, but all lipid classes except LacCer became unstable by day 5. Under refrigerated conditions (4 °C), all lipid classes except GQ1 and Gb3 were stable for up to 7 days. Under frozen conditions (− 20 °C), most lipid classes were stable for up to 14 days (Supplemental Fig. 1). The freeze–thaw stability of all targeted lipid classes was evaluated over four freeze–thaw cycles, with an average recovery rate of 90 to 96% across all cycles, indicating stability (Supplemental Fig. 1). The stability of samples after lipid extraction was assessed in the autosampler at 4 °C, demonstrating stability for up to 24 h (Supplemental Table 5).

**Table 1:** Method validation for multiplexed LC–MS/MS assay

Targeted species	Limit of detection	*R*^2^	QC	Intra-assay (*n* = 5)		Inter-assay (*n* = 5)	
				Mean ± SD	CV%	Mean ± SD	CV%
GM1 (d18:1/18:0)	298 amol	0.995	Low	0.045 ± 0.001	2.53%	0.050 ± 0.006	12.24%
			High	5.41 ± 0.18	3.46%	5.12 ± 0.24	4.68%
GM2 (d18:1/18:0)	321 amol	0.996	Low	0.050 ± 0.002	3.55%	0.047 ± 0.003	6.37%
			High	6.04 ± 0.47	7.91%	5.98 ± 0.84	14.05%
GM3 (d18:1/18:0)	134 amol	0.995	Low	0.052 ± 0.002	1.89%	0.041 ± 0.002	4.75%
			High	5.10 ± 0.11	2.08%	5.50 ± 0.16	2.82%
GD3 (d18:1/18:0)	249 amol	0.998	Low	0.045 ± 0.003	4.94%	0.054 ± 0.009	17.04%
			High	6.50 ± 0.35	5.49%	6.72 ± 0.39	5.80%
GD2 (d18:1/18:0)	1054 amol	0.994	Low	0.048 ± 0.003	6.80%	0.049 ± 0.002	4.11%
			High	6.56 ± 0.30	4.61%	6.31 ± 0.41	6.49%
GD1a (d18:1/18:0)	576 amol	0.993	Low	0.048 ± 0.004	8.24%	0.051 ± 0.003	5.95%
			High	4.15 ± 0.03	0.66%	4.52 ± 0.32	7.07%
GT1b (d18:1/18:0)	188 amol	0.998	Low	0.044 ± 0.004	8.20%	0.050 ± 0.006	11.81%
			High	5.57 ± 0.31	5.66%	5.12 ± 0.45	8.78%

*Abbreviations*: *QC* quality control sample, *SD* standard deviation, *CV* coefficient of variation

### Endogenous targets measured in samples from human serum samples

As shown in Fig. 2B, we employed the multiplexed method to target and analyze various ganglioside and glycosphingolipid species in lipid extracts from serum of individuals. In order to compare the abundance of endogenous gangliosides and other glycosphingolipid species, we analyzed lipid extracts obtained from serum samples from individuals under 20 years of age (ranging from five days to 20 years) and three GM2-gangliosidosis patients. Using the calibration curve derived from the standard mixture spiked into serum substitute (as indicated in Table 1), we quantified the endogenous targets detected in the lipid samples from 36 individuals in the control group and three patients as shown in Table 2. Quantification of the total 7 classes of gangliosides (d36:1) revealed that GM3 ganglioside was the most abundant among the gangliosides present in the serum samples of the 36 individuals in the control group (average 397.4 fmol). Following GM3, GD3 and GM2 were the subsequent most abundant gangliosides (average 235.9 and 203.1 fmol respectively across the 36 individuals).

**Table 2:** Quantitative results of endogenous targets detected in serum from 36 healthy individuals and 3 patients

Groups	Sample ID	Sex	Age	Calculated amount (fmol)						
				GM3 (d18:1/18:0)	GM2 (d18:1/18:0)	GM1 (d18:1/18:0)	GD3 (d18:1/18:0)	GD2 (d18:1/18:0)	GD1 (d18:1/18:0)	GT1 (d18:1/18:0)
Control	CS01	M	3	476.9	372.4	110.3	212.9	173.5	102.9	31.0
	CS02	F	11	497.2	125.6	66.5	109.6	108.5	70.8	19.4
	CS03	M	8	236.3	58.2	26.5	113.4	85.6	63.5	22.5
	CS04	M	13	674.1	124.6	70.7	81.3	122.9	68.0	26.9
	CS05	M	3	368.9	104.8	61.7	170.5	139.5	144.7	19.9
	CS06	F	7	296.1	68.7	32.7	130.8	78.0	70.4	17.4
	CS07	F	4 w	374.6	192.0	57.0	186.5	137.4	104.2	39.9
	CS08	F	4	369.6	107.0	85.7	99.0	98.7	58.4	16.3
	CS09	F	3	400.9	183.3	73.7	184.5	101.7	70.4	20.0
	CS10	M	13	361.3	85.4	60.2	96.1	99.1	69.5	16.6
	CS11	F	9	355.7	58.3	25.7	165.0	157.8	101.2	34.4
	CS12	F	20	327.4	105.2	61.5	163.4	85.2	95.9	24.6
	CS13	F	19	318.4	366.7	44.7	167.7	147.2	76.3	25.0
	CS14	F	2 m	440.5	213.6	65.2	484.9	180.9	298.9	100.9
	CS15	M	3 w	534.2	96.8	41.3	132.8	103.9	84.4	24.6
	CS16	M	20	319.5	145.5	46.3	241.2	155.2	114.3	43.7
	CS17	F	9	405.6	90.3	38.4	303.0	111.6	126.5	51.7
	CS18	F	5 m	492.4	229.3	110.6	175.1	88.3	173.6	54.9
	CS19	M	3	491.4	134.6	94.5	356.8	62.8	154.9	38.7
	CS20	F	3	388.7	578.0	93.9	239.5	110.8	113.3	30.0
	CS21	M	15	402.0	104.8	95.1	240.1	142.1	67.8	33.9
	CS22	M	11	357.9	268.0	159.5	413.6	101.6	213.9	61.8
	CS23	F	9	378.5	193.5	80.4	303.8	41.8	98.7	33.8
	CS24	M	5	297.2	193.7	105.5	289.7	52.3	187.1	58.4
	CS25	F	5 d	452.0	134.0	76.0	112.8	63.3	43.9	14.3
	CS26	M	5	275.4	345.1	170.1	365.5	85.3	190.5	59.7
	CS27	F	16 m	441.1	115.0	71.8	322.2	32.0	109.4	33.3
	CS28	F	3 m	296.6	384.0	136.4	260.3	57.5	201.3	52.3
	CS29	F	26 m	465.1	166.7	117.4	254.3	56.5	196.8	58.9
	CS30	M	17	535.9	124.0	93.4	108.1	33.3	99.2	27.6
	CS31	M	10	297.5	177.6	176.6	162.9	140.4	112.6	27.2
	CS32	F	4 w	355.0	306.2	83.7	323.6	45.3	116.0	38.4
	CS33	M	3	294.0	284.6	215.8	356.1	61.6	126.0	28.9
	CS34	M	8	431.3	381.7	209.8	566.0	76.2	206.5	71.0
	CS35	M	20	429.5	584.6	184.3	283.2	39.3	110.3	31.1
	CS36	M	23	465.9	106.9	36.3	317.3	131.7	122.7	16.5
Patient	TSD01	F	3	251.7	2905.3	464.8	161.2	107.2	85.2	24.0
	TSD02	M	5	224.2	1929.1	627.6	319.3	167.4	190.1	27.8
	SD01	M	7	199.6	1273.1	471.5	221.3	99.2	57.1	16.5

Age in years except where indicated; *d* days, *w* weeks, and *m* months

### Ganglioside alterations in patients with GM2-gangliosidosis

**Fig. 3:**
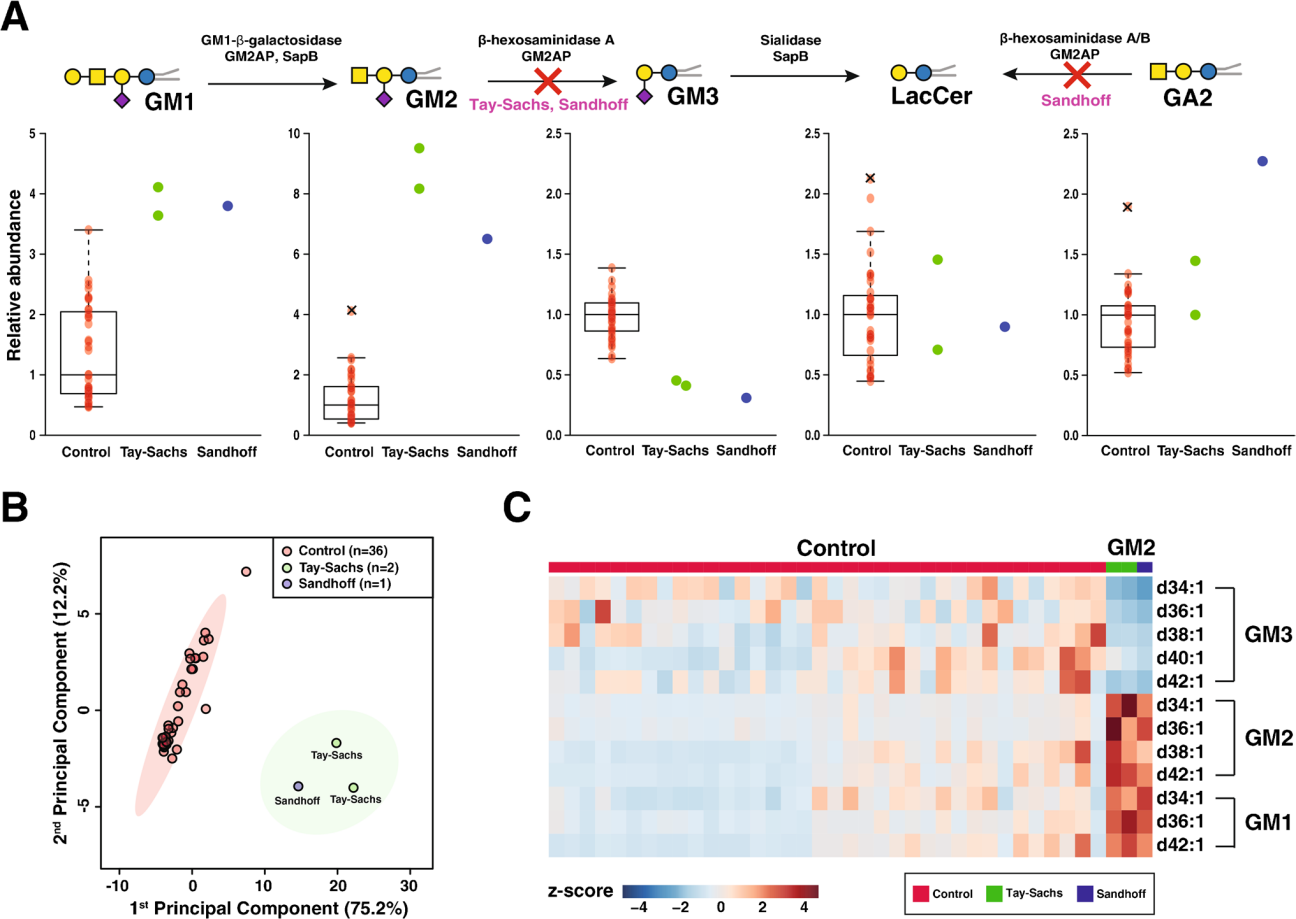
Monosialogangliosides in serum of patients with GM2-gangliosidosis. **A** Box plots showing levels of GM gangliosides (GM1, GM2, and GM3), LacCer, and GA2 in a cohort of 36 control individuals (red circles), 2 Tay-Sachs patients (green circles), and 1 Sandhoff patient (blue circles). The relevant pathway and the enzymes are shown at the top where an X indicates a defect at the corresponding step in the pathway. **B** Principal component analysis plot generated based on the GM abundance levels in GM2-gangliosidosis patients (shown in green) and control individuals (shown in red). **C** A heatmap illustrating the abundance levels of individual GM3, GM2, and GM1 species that show significant differences between the control and GM2-gangliosidosis patient groups

**Fig. 4:**
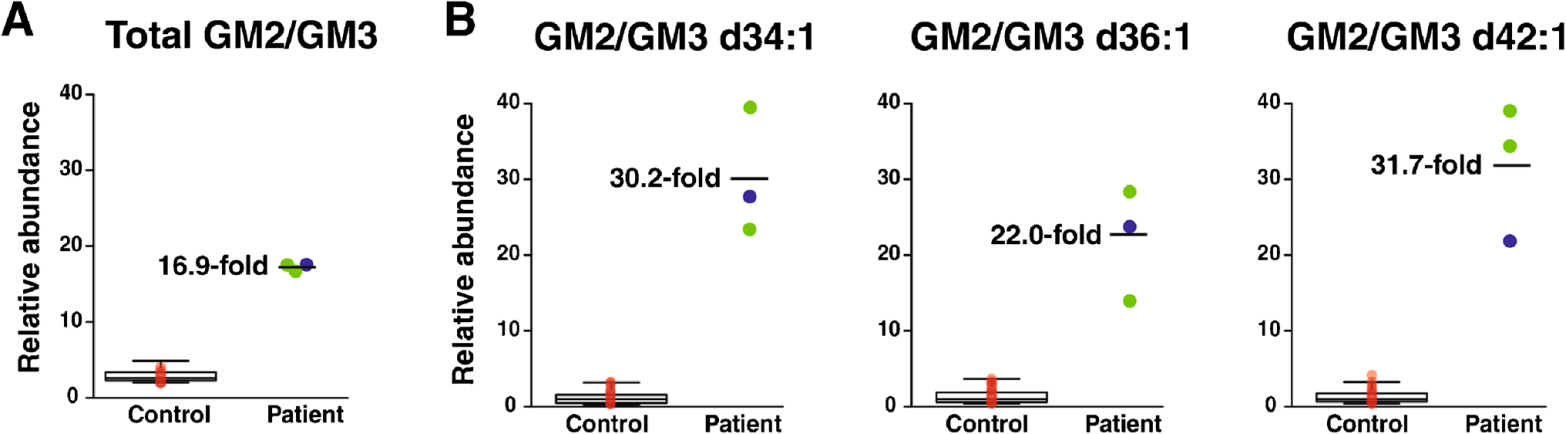
Relative abundance of GM2 to GM3 in GM2-gangliosidosis patients (Tay-Sachs patients, green; Sandhoff patient, blue) and control individuals (red). Box plots of **A** total GM2/GM3 and **B** GM2/GM3 ratios of individual species (d34:1, d36:1 and d42:1) are shown along with the average fold-change indicated in each case

We applied the developed method to investigate whether changes in serum lipids of GM2-gangliosidosis patients (Tay-Sach (*n* = 2) and Sandhoff (*n* = 1)) could be used to distinguish them from controls (*n* = 36). When comparing patients and controls, the total level of GM1 was increased by a median fold-change of ~ 4 in patients (adjusted *p*-values < 0.01) (Fig. 3A). The total levels of GM2 were also increased by a median fold-change of ~ 9 in Tay-Sachs patients and ~ 7 in Sandhoff patients (adjusted *p*-values < 0.01) (Fig. 3A). On the other hand, GM3 levels in the patient group were decreased to 0.3-fold (adjusted *p*-value < 0.01) and the total levels of LacCer showed no statistically significant changes (Fig. 3A). Other targeted gangliosides and glycosphingolipids, except for GA2, showed no statistically significant changes while GA2 was increased 2.4-fold in the patient with Sandhoff disease (Fig. 3A). Principal component analysis (PCA) based on quantitative values of GM1, GM2, and GM3 was generated to visually represent the differential individuals who were tested. The controls could be easily distinguished from patients (Fig. 3B). All GM3 species were decreased with *p*-value < 0.01 in the GM2-gangliosidosis in patients, regardless of the length of the fatty acyl chain. Individual species of GM2 (except for GM2 d40:1) were significantly increased (*p* < 0.01) in patients and three GM1 species (GM1 d34:1, d36:1, and d42:1) also increased significantly (*p* < 0.01) in patients. A heatmap of individual lipid species of GM3, GM2, and GM1 is shown in Fig. 3C. Both Tay-Sachs disease and Sandhoff disease involve accumulation of GM2 and decrease in GM3. Thus, calculating the ratio of GM2/GM3 can further highlight the changes in the GM2-gangliosidosis patient group. In Fig. 4A, the changes in total GM2 to GM3 ratio are presented whereas Fig. 4B shows the fold-changes in the GM2/GM3 ratios for individual species (d34:1, d36:1, and d42:1). While the fold-change in the total ratio of GM2/GM3 was 16.9, the changes in the GM2/GM3 ratios for the individual species d34:1, d36:1, and d42:1 were even more dramatic (30, 22, and 32, respectively).

## Discussion

Various LC–MS/MS methods have been described and applied for quantitative determination of ganglioside molecular species from a range of biological specimens including plasma, cells, and tissues [14, 17–19, 23, 30]. These methods have been used to describe changes in ganglioside levels in diseases associated with corresponding metabolic disorders [16, 17, 19, 31]. These methods have generally focused on measuring specific molecular species of gangliosides or have been applied to various matrices (e.g., fibroblast, leukocyte, plasma, and tissue) other than serum [16, 19, 23]. Thus, there is a lack of an LC–MS/MS-based method that can simultaneously target all gangliosides and glycosphingolipids to provide an integrated view of ganglioside metabolism. In this study, we have developed a robust multiplexed targeted LC–MS/MS method to simultaneously quantify the 84 molecular species of 10 ganglioside classes (GM1, GM2, GM3, GD1, GD2, GD3, GT1, GT2, GT3, and GQ1) and four classes of other glycosphingolipids (GlcCer, LacCer, Gb3, and GA2) in human serum. Using this multiplexed method, we successfully detected 61 of the 84 targeted species of gangliosides and glycosphingolipids in human serum. Additionally, by testing serum samples from individuals with five different ages, we were able to quantify the amounts of endogenous gangliosides from 7 different classes (GM1, GM2, GM3, GD1, GD2, GD3, GT1) using the calibration curve generated from ganglioside standards spiked into serum substitute matrix.

We compared the profiles of gangliosides and other glycosphingolipids in the serum of GM2-gangliosidosis patients (two Tay-Sachs patients and one Sandhoff patient) and 36 age-matched control individuals. The total levels of GM1 and GM2 gangliosides were significantly increased while the levels of GM3 exhibited a significant decrease, in line with other studies on Tay-Sachs and Sandhoff that used fibroblasts or leukocyte samples [19]. While the increased concentration of GM2 d34:1 ganglioside has been reported in plasma of individuals with GM2-gangliosidosis including Tay-Sachs and Sandhoff disease, it has not previously been measured in the serum of patients [14]. Similarly, elevated levels of Lyso-GM2 gangliosides have been identified in the plasma of GM2-gangliosidosis patients, although not consistently in every case [32, 33]. Naturally affected animals afflicted with GM2-gangliosidosis, such as cats, dogs, and sheep, have been studied and the accumulation of GM2 gangliosides and asialo-GM2 gangliosides (GA2) has been observed within the brain and various tissues [34, 35]. However, to the best of our current knowledge, there has been no reports describing changes in ganglioside levels in serum samples from GM2-gangliosidosis patients. Sandhoff disease is characterized by the accumulation of GM2 ganglioside due to deficiency of both β-hexosaminidases A and B in contrast to Tay-Sachs disease in which only β-hexosaminidase A deficiency occurs. Specifically, the increased levels of GA2 in Sandhoff disease can be attributed to the deficiency of β-hexosaminidase B. β-Hexosaminidase B is responsible for the degradation of GA2 to convert it into GM2 ganglioside. Notably, in Sandhoff disease, the deficiency of β-hexosaminidase B also leads to the accumulation of GA2 [11]. For this reason, the levels of GA2 are elevated in with the patient with Sandhoff disease in this study. Upon quantification of individual species, GM2 d34:1 and GM2 d36:1 exhibited a substantial increase (10.3-fold and 10.0-fold, respectively) in GM2-gangliosidosis patients as compared to controls, whereas GM3 d34:1 and GM3 d42:1 were significantly decreased (0.37-fold and 0.23-fold, respectively) in the patient group. Since β-hexosaminidase A is responsible for the hydrolysis of GM2 to GM3, the deficiency of this enzyme in Tay-Sachs and Sandhoff diseases is anticipated to result in the accumulation of GM2 and decrease of GM3. The ratio of total and individual species of GM2/GM3 ratio were elevated in patients compared to controls. These ratios have the potential to serve as markers for GM2-gangliosidosis. Our data support previous reports [14, 18] of GM2 d34:1 as the most distinguishing molecular species between GM2-gangliosidosis patients and controls, emphasizing the significance of measuring individual species rather than total GM2 ganglioside levels.

## Conclusions

Our study presents a multiplexed targeted LC–MS/MS method for quantifying gangliosides and glycosphingolipids in human serum. In GM2-gangliosidosis patients, total GM1 and GM2 levels are elevated with a concomitant decrease in GM3 levels. Notably, the ratios of GM2/GM3 d34:1 and d42:1 show a substantial increase in patients. This approach showcases the potential utility of this method to screen and/or diagnose rare genetic disorders involving changes in ganglioside metabolism.

## Supplementary Information

Below is the link to the electronic supplementary material.

Supplementary file1 (DOCX 165 KB)

## Data Availability

The data that support the findings of this study are available on request from the corresponding author.
