# Supplementary material for: A multiplexed targeted method for profiling of serum gangliosides and glycosphingolipids: application to GM2-gangliosidosis
Source: Anal Bioanal Chem. 2024 Aug 27;416(26):5689–99. doi: 10.1007/s00216-024-05487-3 (PMC11493836; doi:10.1007/s00216-024-05487-3)

**Supplementary Information:**

**A Multiplexed Targeted Method for Profiling of Serum Gangliosides and Glycosphingolipids: Application to GM2-gangliosidosis**

Jinyong Kim^1^, Seul Kee Byeon^1^, Devin Oglesbee^1^, Matthew J. Schultz^1^, Dietrich Matern^1^, Akhilesh Pandey^1,2,3,*^

^1^Department of Laboratory Medicine and Pathology, Mayo Clinic, Rochester, Minnesota, USA

^2^Center for Individualized Medicine, Mayo Clinic, Rochester, Minnesota, USA

^3^Manipal Academy of Higher Education, Manipal, Karnataka, India

*Corresponding author:

Akhilesh Pandey

200 First Street SW, Rochester, MN 55905, USA

E-mail address: [pandey.akhilesh@mayo.edu](mailto:pandey.akhilesh@mayo.edu)

Supplementary Table 1. patient with GM2 gangliosidosis and 36 controls

| Groups | Sample ID | Sex | Age |
| --- | --- | --- | --- |
| Patient | TSD01 | F | 3 |
|  | TSD02 | M | 5 |
|  | SD01 | M | 7 |
| Control | CS01 | M | 3 |
|  | CS02 | F | 11 |
|  | CS03 | M | 8 |
|  | CS04 | M | 13 |
|  | CS05 | M | 3 |
|  | CS06 | F | 7 |
|  | CS07 | F | 4 w |
|  | CS08 | F | 4 |
|  | CS09 | F | 3 |
|  | CS10 | M | 13 |
|  | CS11 | F | 9 |
|  | CS12 | F | 20 |
|  | CS13 | F | 19 |
|  | CS14 | F | 2 m |
|  | CS15 | M | 3 w |
|  | CS16 | M | 20 |
|  | CS17 | F | 9 |
|  | CS18 | F | 5 m |
|  | CS19 | M | 3 |
|  | CS20 | F | 3 |
|  | CS21 | M | 15 |
|  | CS22 | M | 11 |
|  | CS23 | F | 9 |
|  | CS24 | M | 5 |
|  | CS25 | F | 5 d |
|  | CS26 | M | 5 |
|  | CS27 | F | 16 m |
|  | CS28 | F | 3 m |
|  | CS29 | F | 26 m |
|  | CS30 | M | 17 |
|  | CS31 | M | 10 |
|  | CS32 | F | 4 w |
|  | CS33 | M | 3 |
|  | CS34 | M | 8 |
|  | CS35 | M | 20 |
|  | CS36 | M | 13 |

*Age in years except where indicated; d - days, w - weeks and m – months

*TSD – Tay-Sachs disease, SD – Sandhoff disease, CS – control serum

Supplementary Table 2. LC-MS/MS parameters of the targeted gangliosides, glycosphingolipids and internal standards

| Compound | Precursor (m/z) | Product (m/z) | Collision Energy (V) | Retention time (min) | RT window (min) | Polarity | Assigned IS |
| --- | --- | --- | --- | --- | --- | --- | --- |
| GM3 C16:0 | 1151.7 | 290.1 | -80 | 5.18 | 1 | negative | GM3 C18:0-d5 |
| GM3 C18:0 | 1179.7 | 290.1 | -80 | 5.32 | 1 | negative | GM3 C18:0-d5 |
| GM3 C20:0 | 1207.7 | 290.1 | -80 | 5.55 | 1 | negative | GM3 C18:0-d5 |
| GM3 C22:0 | 1235.8 | 290.1 | -80 | 5.82 | 1 | negative | GM3 C18:0-d5 |
| GM3 C24:0 | 1263.8 | 290.1 | -80 | 5.96 | 1 | negative | GM3 C18:0-d5 |
| GM3 C24:1 | 1261.8 | 290.1 | -80 | 5.89 | 1 | negative | GM3 C18:0-d5 |
| GM2 C16:0 | 1354.7 | 290.1 | -80 | 5.18 | 1 | negative | GM3 C18:0-d5 |
| GM2 C18:0 | 1382.8 | 290.1 | -80 | 5.32 | 1 | negative | GM3 C18:0-d5 |
| GM2 C20:0 | 1410.8 | 290.1 | -80 | 5.55 | 1 | negative | GM3 C18:0-d5 |
| GM2 C22:0 | 1438.8 | 290.1 | -80 | 5.82 | 1 | negative | GM3 C18:0-d5 |
| GM2 C24:0 | 1466.9 | 290.1 | -80 | 5.96 | 1 | negative | GM3 C18:0-d5 |
| GM2 C24:1 | 1464.9 | 290.1 | -80 | 5.9 | 1 | negative | GM3 C18:0-d5 |
| GM1 C16:0 | 1516.8 | 290.1 | -80 | 5.18 | 1 | negative | GM1 C17:0 |
| GM1 C18:0 | 1544.8 | 290.1 | -80 | 5.32 | 1 | negative | GM1 C17:0 |
| GM1 C20:0 | 1572.9 | 290.1 | -80 | 5.55 | 1 | negative | GM1 C17:0 |
| GM1 C22:0 | 1600.9 | 290.1 | -80 | 5.82 | 1 | negative | GM1 C17:0 |
| GM1 C24:0 | 1628.9 | 290.1 | -80 | 5.96 | 1 | negative | GM1 C17:0 |
| GM1 C24:1 | 1626.9 | 290.1 | -80 | 5.9 | 1 | negative | GM1 C17:0 |
| GD3 C16:0 | 720.8 | 290.1 | -45 | 4.59 | 1 | negative | GM1 C17:0 |
| GD3 C18:0 | 734.9 | 290.1 | -45 | 4.68 | 1 | negative | GM1 C17:0 |
| GD3 C20:0 | 748.9 | 290.1 | -45 | 4.77 | 1 | negative | GM1 C17:0 |
| GD3 C22:0 | 762.9 | 290.1 | -45 | 4.86 | 1 | negative | GM1 C17:0 |
| GD3 C24:0 | 776.9 | 290.1 | -45 | 5.01 | 1 | negative | GM1 C17:0 |
| GD3 C24:1 | 774.9 | 290.1 | -45 | 4.95 | 1 | negative | GM1 C17:0 |
| GD2 C16:0 | 822.4 | 290.1 | -45 | 4.59 | 1 | negative | GM1 C17:0 |
| GD2 C18:0 | 836.4 | 290.1 | -45 | 4.68 | 1 | negative | GM1 C17:0 |
| GD2 C20:0 | 850.4 | 290.1 | -45 | 4.77 | 1 | negative | GM1 C17:0 |
| GD2 C22:0 | 864.4 | 290.1 | -45 | 4.86 | 1 | negative | GM1 C17:0 |
| GD2 C24:0 | 878.4 | 290.1 | -45 | 5.01 | 1 | negative | GM1 C17:0 |
| GD2 C24:1 | 876.4 | 290.1 | -45 | 4.95 | 1 | negative | GM1 C17:0 |
| GD1 C16:0 | 903.4 | 290.1 | -45 | 4.59 | 1 | negative | GM1 C17:0 |
| GD1 C18:0 | 917.4 | 290.1 | -45 | 4.68 | 1 | negative | GM1 C17:0 |
| GD1 C20:0 | 931.4 | 290.1 | -45 | 4.77 | 1 | negative | GM1 C17:0 |
| GD1 C22:0 | 945.5 | 290.1 | -45 | 4.86 | 1 | negative | GM1 C17:0 |
| GD1 C24:0 | 959.5 | 290.1 | -45 | 5.01 | 1 | negative | GM1 C17:0 |
| GD1 C24:1 | 957.5 | 290.1 | -45 | 4.95 | 1 | negative | GM1 C17:0 |
| GT3 C16:0 | 866.4 | 290.1 | -50 | 4.36 | 1 | negative | GM1 C17:0 |
| GT3 C18:0 | 880.4 | 290.1 | -50 | 4.45 | 1 | negative | GM1 C17:0 |
| GT3 C20:0 | 894.4 | 290.1 | -50 | 4.54 | 1 | negative | GM1 C17:0 |
| GT3 C22:0 | 908.4 | 290.1 | -50 | 4.59 | 1 | negative | GM1 C17:0 |
| GT3 C24:0 | 922.5 | 290.1 | -50 | 4.67 | 1 | negative | GM1 C17:0 |
| GT3 C24:1 | 920.5 | 290.1 | -50 | 4.63 | 1 | negative | GM1 C17:0 |
| GT2 C16:0 | 967.9 | 290.1 | -50 | 4.36 | 1 | negative | GM1 C17:0 |
| GT2 C18:0 | 981.9 | 290.1 | -50 | 4.45 | 1 | negative | GM1 C17:0 |
| GT2 C20:0 | 996 | 290.1 | -50 | 4.54 | 1 | negative | GM1 C17:0 |
| GT2 C22:0 | 1010 | 290.1 | -50 | 4.59 | 1 | negative | GM1 C17:0 |
| GT2 C24:0 | 1024 | 290.1 | -50 | 4.67 | 1 | negative | GM1 C17:0 |
| GT2 C24:1 | 1022 | 290.1 | -50 | 4.63 | 1 | negative | GM1 C17:0 |
| GT1 C16:0 | 1049 | 290.1 | -50 | 4.36 | 1 | negative | GM1 C17:0 |
| GT1 C18:0 | 1063 | 290.1 | -50 | 4.45 | 1 | negative | GM1 C17:0 |
| GT1 C20:0 | 1077 | 290.1 | -50 | 4.54 | 1 | negative | GM1 C17:0 |
| GT1 C22:0 | 1091 | 290.1 | -50 | 4.59 | 1 | negative | GM1 C17:0 |
| GT1 C24:0 | 1105 | 290.1 | -50 | 4.67 | 1 | negative | GM1 C17:0 |
| GT1 C24:1 | 1103 | 290.1 | -50 | 4.63 | 1 | negative | GM1 C17:0 |
| GQ1 C16:0 | 1194.5 | 290.1 | -50 | 4.02 | 1 | negative | GM1 C17:0 |
| GQ1 C18:0 | 1208.5 | 290.1 | -50 | 4.09 | 1 | negative | GM1 C17:0 |
| GQ1 C20:0 | 1222.5 | 290.1 | -50 | 4.17 | 1 | negative | GM1 C17:0 |
| GQ1 C22:0 | 1236.6 | 290.1 | -50 | 4.27 | 1 | negative | GM1 C17:0 |
| GQ1 C24:0 | 1250.6 | 290.1 | -50 | 4.4 | 1 | negative | GM1 C17:0 |
| GQ1 C24:1 | 1248.6 | 290.1 | -50 | 4.33 | 1 | negative | GM1 C17:0 |
| LacCer C16:0 | 862.6 | 264.3 | 80 | 6.99 | 1 | positive | Gb3 C18:0-d3 |
| LacCer C18:0 | 890.7 | 264.3 | 80 | 7.19 | 1 | positive | Gb3 C18:0-d3 |
| LacCer C20:0 | 918.7 | 264.3 | 80 | 7.38 | 1 | positive | Gb3 C18:0-d3 |
| LacCer C22:0 | 946.7 | 264.3 | 80 | 7.54 | 1 | positive | Gb3 C18:0-d3 |
| LacCer C24:0 | 974.8 | 264.3 | 80 | 7.7 | 1 | positive | Gb3 C18:0-d3 |
| LacCer C24:1 | 972.7 | 264.3 | 80 | 7.61 | 1 | positive | Gb3 C18:0-d3 |
| GlcCer C16:0 | 700.6 | 264.3 | 80 | 7.08 | 1 | positive | GlcCer C16:0-d3 |
| GlcCer C18:0 | 728.6 | 264.3 | 80 | 7.26 | 1 | positive | GlcCer C16:0-d3 |
| GlcCer C20:0 | 756.6 | 264.3 | 80 | 7.43 | 1 | positive | GlcCer C16:0-d3 |
| GlcCer C22:0 | 784.7 | 264.3 | 80 | 7.6 | 1 | positive | GlcCer C16:0-d3 |
| GlcCer C24:0 | 812.7 | 264.3 | 80 | 7.75 | 1 | positive | GlcCer C16:0-d3 |
| GlcCer C24:1 | 810.7 | 264.3 | 80 | 7.67 | 1 | positive | GlcCer C16:0-d3 |
| Gb3 C16:0 | 1024.7 | 264.3 | 80 | 6.94 | 1 | positive | Gb3 C18:0-d3 |
| Gb3 C18:0 | 1052.7 | 264.3 | 80 | 7.13 | 1 | positive | Gb3 C18:0-d3 |
| Gb3 C20:0 | 1080.7 | 264.3 | 80 | 7.32 | 1 | positive | Gb3 C18:0-d3 |
| Gb3 C22:0 | 1108.8 | 264.3 | 80 | 7.52 | 1 | positive | Gb3 C18:0-d3 |
| Gb3 C24:0 | 1136.8 | 264.3 | 80 | 7.7 | 1 | positive | Gb3 C18:0-d3 |
| Gb3 C24:1 | 1134.8 | 264.3 | 80 | 7.57 | 1 | positive | Gb3 C18:0-d3 |
| GA2 C16:0 | 1065.7 | 264.3 | 80 | 6.9 | 1 | positive | GlcCer C16:0-d3 |
| GA2 C18:0 | 1093.7 | 264.3 | 80 | 7.1 | 1 | positive | GlcCer C16:0-d3 |
| GA2 C20:0 | 1121.8 | 264.3 | 80 | 7.48 | 1 | positive | GlcCer C16:0-d3 |
| GA2 C22:0 | 1149.8 | 264.3 | 80 | 7.6 | 1 | positive | GlcCer C16:0-d3 |
| GA2 C24:0 | 1177.8 | 264.3 | 80 | 7.75 | 1 | positive | GlcCer C16:0-d3 |
| GA2 C24:1 | 1175.8 | 264.3 | 80 | 7.62 | 1 | positive | GlcCer C16:0-d3 |
| GM3 C18:0-d5 | 1184.7 | 290.1 | -80 | 5.35 | 1 | negative | - |
| GM1 C17:0 | 1530.8 | 290.1 | -80 | 5.24 | 1.5 | negative | - |
| GlcCer C16:0-d3 | 701.6 | 264.3 | 80 | 7.08 | 1 | positive | - |
| Gb3 C18:0-d3 | 1055.7 | 264.3 | 80 | 7.14 | 1 | positive | - |

Supplemental table 3. Extraction efficiency (%) of gangliosides and complex sphingolipids using chloroform:methanol (1:2, v/v) as extraction solvent.

| Lipid species | Extraction efficiency (%; n=3) |
| --- | --- |
| GM1 | 99.7 ± 6.9 |
| GM2 | 109.5 ± 6.2 |
| GM3 | 99.6 ± 9.6 |
| GD1 | 98.2 ± 6.8 |
| GD2 | 102.9 ± 6.3 |
| GD3 | 99.3 ± 7.3 |
| GT1 | 98.8 ± 7.6 |
| GA2 | 100.0 ± 7.5 |
| Hex1Cer (GlcCer) | 96.1 ± 7.7 |
| Hex2Cer (LacCer) | 100.1 ± 9.8 |
| Hex3Cer (Gb3) | 98.1 ± 3.1 |

Supplemental table 4. Matrix effect (%) of low (50 fmol/µl) mixture and high (5 pmol/µl) mixture of ganglioside standards spiked to lipid-free serum.

| **Targeted species** | **Sample** | **Matrix effect (%; n=3)** |  |
| --- | --- | --- | --- |
|  |  |  |  |
| GM1 (d18:1/18:0) | low mix | 78.5 ± 6.2 |  |
|  | high mix | 85.4 ± 3.8 |  |
| GM2 (d18:1/18:0) | low mix | 80.5 ± 5.2 |  |
|  | high mix | 86.4 ± 4.7 |  |
| GM3 (d18:1/18:0) | low mix | 90.2 ± 2.3 |  |
|  | high mix | 95.1 ± 6.1 |  |
| GD3 (d18:1/18:0) | low mix | 84.5 ± 3.8 |  |
|  | high mix | 93.5 ± 3.5 |  |
| GD2 (d18:1/18:0) | low mix | 76.2 ± 8.3 |  |
|  | high mix | 80.6 ± 4.3 |  |
| GD1a (d18:1/18:0) | low mix | 67.4 ± 4.4 |  |
|  | high mix | 81.2 ± 7.3 |  |
| GT1b (d18:1/18:0) | low mix | 79.4 ± 4.9 |  |
|  | high mix | 90.5 ± 5.1 |  |

Supplemental table 5. Autosampler post-extraction stability test. Stability of lipid extracts kept inside autosampler (4°C, 48 hours) was assessed by injecting lipid extracts over 48 hours. Recovery (%) in lipid classes as compared to time zero point are summarized.

| Lipid classes | Recovery (%) vs. baseline (time 0) | | | | | |
| --- | --- | --- | --- | --- | --- | --- |
|  | 4 hrs | 8 hrs | 12 hrs | 16 hrs | 24 hrs | 48 hrs |
| GM3 | 98.6 | 103.6 | 99.6 | 93.3 | 86.4 | 80.1 |
| GM2 | 105.6 | 110.2 | 102.6 | 95.6 | 90.1 | 81.6 |
| GM1 | 98.4 | 98.3 | 95.7 | 87.1 | 88.4 | 80.4 |
| GD3 | 90.4 | 106.0 | 94.8 | 89.9 | 88.4 | 81.8 |
| GD2 | 109.8 | 108.9 | 109.6 | 90.4 | 94.0 | 82.3 |
| GD1 | 108.7 | 109.7 | 108.7 | 95.6 | 91.7 | 74.7 |
| GT3 | 89.5 | 97.4 | 99.0 | 94.7 | 91.2 | 84.9 |
| GT2 | 88.9 | 107.8 | 106.0 | 95.6 | 88.3 | 74.6 |
| GT1 | 98.3 | 105.0 | 101.3 | 89.5 | 86.8 | 80.8 |
| GQ1 | 95.4 | 95.0 | 92.2 | 93.6 | 88.7 | 81.6 |
| GlcCer | 95.4 | 92.5 | 97.2 | 92.6 | 89.9 | 71.3 |
| LacCer | 93.9 | 107.3 | 104.2 | 95.0 | 93.6 | 82.6 |
| Gb3 | 97.1 | 97.7 | 111.6 | 93.9 | 88.1 | 84.3 |
| GA2 | 96.6 | 103.7 | 96.5 | 91.3 | 90.4 | 81.3 |

Supplemental figure 1. Evaluation of sample stability. The variation in targeted lipid classes compared with baseline concentrations (0 d or 0 cycle) during storage at (A) room temperature, (B) 4 °C (refrigerated), (C) -20 °C (frozen) and (D) freeze-thaw cycles (up to 4 cycles). The red dotted lines indicate the acceptable range (85% to 115%)


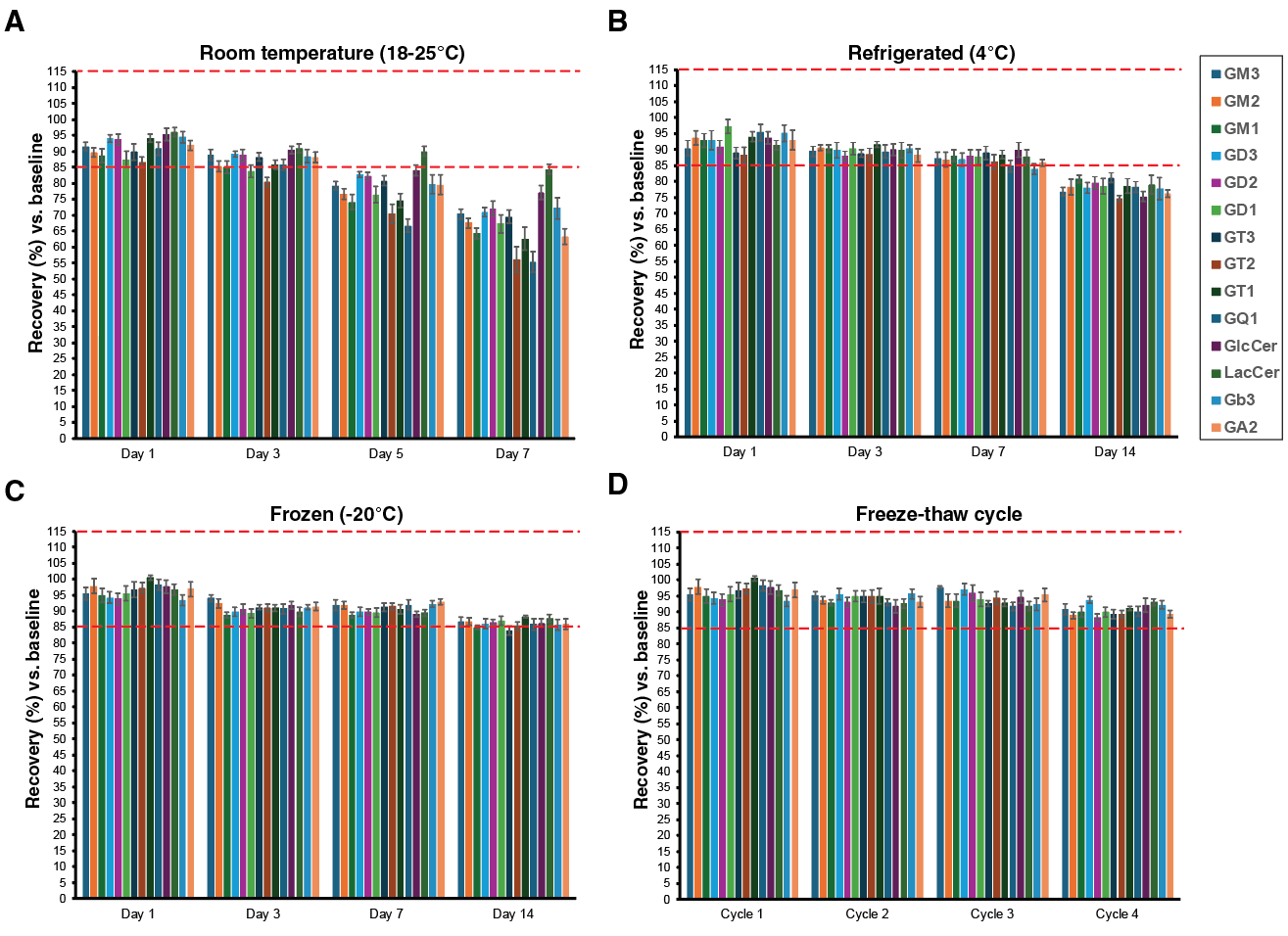

Supplement: Supplementary file 1 — Supplementary file1 (DOCX 165 KB) [file 216_2024_5487_MOESM1_ESM.docx]
